# Comparing Students of Medical and Social Sciences in Terms of Self-Assessment of Perceived Stress, Quality of Life, and Personal Characteristics

**DOI:** 10.3389/fpsyg.2022.815369

**Published:** 2022-04-13

**Authors:** Magda K. Wielewska, Julia M. Godzwon, Kacper Gargul, Emma Nawrocka, Kinga Konopka, Krzysztof Sobczak, Agata Rudnik, Agata Zdun-Ryzewska

**Affiliations:** ^1^Department of Social Sciences, Institute of Psychology, University of Gdańsk, Gdańsk, Poland; ^2^Department of Sociology of Medicine and Social Pathology, Faculty of Health Sciences, Medical University of Gdańsk, Gdańsk, Poland; ^3^Department of Gastroenterology, Independent Public Health Care of the Ministry of the Internal Affairs in Gdańsk, Gdańsk, Poland; ^4^Academic Psychological Support Center, University of Gdańsk, Gdańsk, Poland; ^5^Department of Quality of Life Research, Faculty of Health Sciences, Medical University of Gdańsk, Gdańsk, Poland

**Keywords:** mental health, academic performance, students, quality of life, personality

## Abstract

The aim of this study was to compare medical and social sciences students’ outcomes in terms of self-perceived stress, quality of life, and personality traits. We put particular emphasis on external and internal differences in students of specific fields–medicine, nursing, psychology, and pedagogy. In a survey, 1,783 students from Medical University of Gdańsk and University of Gdańsk participated in our study, of whom 1,223 were included in the final statistical analysis. All of them were evaluated using valid and reliable questionnaires–TIPI-PL, PSS-10, and a one-item scale of quality of life. Stress turned out to have a negative effect on quality of life, regardless of the type of field of study. Moreover, students from different fields varied in terms of personality factors: conscientiousness, agreeableness, openness to experience, and emotional stability. In conclusion, many students regardless of their field suffer from high stress and report low quality of life, which potentially further affects their academic performance and social life.

## Introduction

Medical studies, due to an enormous amount of required knowledge, are widely considered to be difficult. Additionally, high social status and responsibility associated with being a medical doctor can make the environment of a medical university a great source of stress. What is more, medical universities are often described by students as full of unhealthy competition (Yusoff, 2014) especially during the first half of medical studies (Midtgaard et al., 2008). Scientists report an immense influence of continuous and excessive stress on physical and mental health (Ribeiro et al., 2018) as well as psychological condition–it may increase the risk of depression, anxiety, and burnout (Pacheco et al., 2017; Pawlaczyk et al., 2020). As a consequence, medical students may be more likely to develop these conditions than the general population (Pacheco et al., 2017). What is more, distress can impact not only their mental health and quality of life but its consequences can also affect their professional functioning, such as academic performance (Dyrbye et al., 2005) and medical career, accounting for poorer patient care and risk of more frequent medical mistakes (Fahrenkopf et al., 2008; De Vibe et al., 2013). Additionally, a similar issue is observed in student nurses population (Chernomas and Shapiro, 2013; Kleiveland et al., 2015). Numerous studies indicate that precisely clinical experience is the most anxiety-inducing part of their education program (Jimenez et al., 2010; Chernomas and Shapiro, 2013) and faced with those difficulties student nurses feel pressured and exhausted (Evans and Kelly, 2004).

However, the presented problem occurs also in other, non-medical professions (who also prepare to work in jobs related to health and psychological condition) and can be observed earlier in the students’ population too. Despite their work and studies not being viewed as stressful by society, psychology (Myers et al., 2012; De Vibe et al., 2013), and pedagogy (Rieg et al., 2007; Rowicka, 2020) students and professionals are also exposed to continuous and excessive stress. Studies on psychologists show that professionals who do not manage job-related stress well are at risk of burnout and impairment (Norcross and VandenBos, 2018). Possibly it would also affect their patients as a psychologists’ health influences their ability to provide professional help (American Psychological Association, 2017).

Although the issue of self-perceived stress, quality of life (QoL), and personality traits in students of medicine and psychology is addressed by researchers, there is also a need to investigate these aspects among students of related fields, such as nursing and pedagogy. Despite the fact that the problems described in earlier paragraphs were investigated in some studies focused on medical students (Henning et al., 2012; Pagnin and De Queiroz, 2015), there is a lack of consensus in the research community about the final shape of relationship between QoL and stress in medicine students, but also in students of social sciences. What is more, there are few studies discussing and comparing the functioning of Polish medical and social field students in the presented issues.

The study carried out by Humburg (2017) suggests that personality traits are as important as cognitive skills when it comes to choosing a university field. For example, conscientiousness is positively related to probability of studying medical sciences (Humburg, 2017). The 6-year longitudinal study conducted by Tyssen et al. (2007) among Norwegian medical students, showed that the combination of both high neuroticism and high conscientiousness can predict medical school stress. Ebstrup et al. (2011) measured the relationship between perceived stress and the NEO Five-Factor Inventory (NEO-FFI) and demonstrated that stress significantly positively correlates with neuroticism and shows a moderate significant negative association with extraversion. Knowing that students of medicine, nursery, and psychology report a higher level of perceived stress, it could be assumed that a many of them will be characterized by a higher level of neuroticism compared to students of pedagogy.

Therefore, the aim of this study was to examine the relationship between the level of stress and QoL in a group of students from varying fields (H1). Moreover, we wanted to explore differences in terms of personality traits among students of difference fields of study (H2). Lastly, we hypothesize medical students in comparison to non–medical students will declare higher stress levels (H3).

## Materials and Methods

### Study Design

Our research focused on finding connections between the perceived level of stress, QoL, and field of study. Personality traits were also measured to control their relationship with variables stated above. We utilized a cross-sectional design and convenience sampling.

### Setting

Study was conducted in the Faculty of Social Sciences at University of Gdańsk and in the Faculty of Medicine and the Faculty of Health Sciences with Institute of Marine and Tropical Medicine at Medical University of Gdańsk from October to December 2019. The aforementioned universities are the only public educational facilities providing studies in the explored fields in the Pomeranian voivodeship. Students who voluntarily agreed to participate in the study were invited to anonymously complete the questionnaires after lectures and other classes on campus. Before the beginning they were notified about the aim of the study and its methodology. Respondents were informed that their answers will be processed in the form of collective statistical analyses and their sensitive or personal data will be neither collected, nor processed. The participants were allowed to resign from further participation in this project at any point of its duration. Respondents were made aware that participation in the study is voluntary and filling out the questionnaire is identical with consent to participate. Typical time of completing the questionnaire varied between 15 and 30 min.

### Participants

There were overall 1,783 participants, which represent seven fields of study in this research project. For this particular paper participants from four fields of study were selected, leaving out 478 participants from other fields of study to avoid surplus of hypotheses in our research article. Moreover, due to errors and omitted answers some participants were excluded, resulting in the final sample consisting of 1,223 students. Mean age in the final sample was 21.37 years, with 79.8% female respondents. The high percentage of female respondents is true to students’ population structure in analyzed fields. In a survey, 54.2% of participants were from Medical University of Gdańsk.

### Variables

Variables included in this study were gender, age, type of university, field of study, year of study, perceived level of stress, QoL, Big Five personality traits. Considering the lack of replies to individual items of the questionnaires, participants with missing or incorrectly provided crucial data were excluded from the specific analysis but included in other analyses. The missing data are: 13 participants did not provide gender, 6 people did not provide any personality subscales, 4 participants did not provide age, 2 people did not provide the year of study, 2 participants did not provide quality of life scale, 5 people did not have a result from the scale stress and some of them did not fulfill more than one item (min 2 and max 3). Hence, we removed these participants from the analysis requiring the aforementioned variables.

### Tools of Measurement

#### Stress

Polish adaptation of Cohen Perceived Stress Scale PSS-10 (Juczyński and Ogińska-Bulik, 2009) was used. This tool includes ten self-assessment items on a scale from 0 (never) to 4 (very often) which identify the level of perceived stress related to the current life situation over the last month. The scale obtained satisfactory internal reliability (α = 0.86) (Juczyński and Ogińska-Bulik, 2009).

#### Quality of Life

Measured using a one-item scale exploring students’ quality of life. Participants answered on a 10-point Likert scale from 1 (not at all satisfied) to 10 (extremely satisfied). It was based on the Cantril Scale (CS) primarily because it allowed us to minimize the number of questions (participants completed the paper-and-pencil test), and also due to the fact it is validated and recognized by researchers. For almost 60 years the Cantril Scale (CS) has been cited as being an effective tool for measuring general well-being, mental health, and happiness. Moreover, the administration of the Cantril Ladder is simple and does not require a major investment of time for either respondent or interviewer (Levin and Currie, 2014; Mazur et al., 2018).

#### Personality

Personality traits were measured with the Short Personality Inventory TIPI-PL (Sorokowska et al., 2014). Questionnaire measures the Big Five personality traits by means of self-description, which the subject performs using a scale of responses from 1 (strongly disagree) to 7 (strongly agree). The authors of a Polish version of the questionnaire—TIPI-PL conducted seven studies that involved a total of 1,772 Polish students and stated the reliability and validity of the Polish version of the TIPI scale were satisfactory. For the paper-and-pencil version of the questionnaire Cronbach-alphas ranged between 0.44 (Openness to Experience) and 0.75 (Conscientiousness), test-retest reliability ranged between 0.56 for Openness to Experience and 0.83 (Emotional stability and Conscientiousness). Correlations with NEO-FFI scales ranged between 0.49 (Openness to Experience) and 0.74 (Conscientiousness). Correlations with NEO-FFI scales ranged between 0.49 (Openness to Experience) and 0.74 (Conscientiousness). In the summary, the scientist underlined that “although TIPI-PL should not be used for in-depth diagnosis, the scale seems to be perfect for scientific research” (Sorokowska et al., 2014).

### Statistical Analysis

Data was analyzed using IBM SPSS Statistics, version 25.0. To examine the associations between stress and other factors, Pearson correlation coefficients were calculated. Analysis of variance was conducted to evaluate personality differences between students from different fields of study, utilizing one way ANOVA with Tukey’s B (for extraversion, emotional stability, and openness to experience) and Tamhane’s T2 (for agreeableness, conscientiousness) *post hoc* tests applied. We performed two different *post hoc* tests for the reason that the assumption of homogeneity of variances has been violated. Statistical comparison of mean reported level of stress for both fields was calculated using Student’s *t*-test for independent samples. All hypotheses were verified at the significance level of *p* < 0.05.

## Results

### Descriptive Statistics

Table 1 presents number of participants in different groups.

**TABLE 1:** Characteristics of the respondents.

Participants	N (% of n)*
**Gender**	
Female	976 (79.8)
Male	234 (19.1)
**Age**	
18–20	433 (35.4)
21–23	605 (49.5)
>0.24	180 (14.7)
**Type of university and field of study**	
Medical university	663 (54.2)
Medical	414 (33.9)
Nursing	249 (20.4)
Non-medical University	560 (45.8)
Psychology	326 (26.7)
Pedagogy	234 (19.1)
**Year of study**	
First	317 (25.9)
Second	218 (17.8)
Third	228 (18.6)
Fourth	234 (19.1)
Fifth	224 (18.3)

*Numbers may not sum to N = 1223 or 100% due to participants not providing answers.

### Analysis of Correlations

Correlational analysis revealed significant negative connections between the perceived level of stress and almost all other variables. QoL did not show significant correlation with field of study (medical vs. social). Correlation coefficients between study variables are shown in the Table 2.

**TABLE 2:** Correlation coefficients (Point-Biserial, Pearson product-moment) between study variables.

Variables	1.	2.	3.	4.	5.	6.	7.	8.	9.
1. Gender^a^	–								
2. Age	0.12**	–							
3. Extraversion	−0.10**	0.01	–						
4. Agreeableness	−0.18**	−0.07*	0.14**	–					
5. Conscientiousness	−0.15**	0.00	0.14**	0.18**	–				
6. Emotional stability	0.16**	0.10**	0.38**	0.09**	0.13**	–			
7. Openness to experience	–0.05	–0.03	0.17**	0.10**	−0.08**	0.00	–		
8. Quality of life	−0.06*	0.06*	0.44**	0.15**	0.23**	0.44**	0.10**	–	
9. Stress	−0.11**	−0.14**	−0.26**	−0.16**	−0.16**	−0.51**	–0.05	−0.48**	–
10. Field of study (medical vs. non-medical)	−0.21**	−0.25**	–0.02	0.06*	−0.09**	−0.11**	0.07*	–0.05	0.00

*^a^Point-Biserial correlation coefficients (1 = female, 2 = male).*

*p < 0.05;**p < 0.01.

### Analysis of Variance

Analysis of variance showed significant personality differences between outcomes of students from different fields of study. Regarding extraversion, psychology students scored significantly lower than nursing and pedagogy students [*F*_(3_,_1215)_ = 4.237; *p* = 0.005]. As for agreeableness, medical students’ results were the highest and differed significantly form nursing and pedagogy students’ outcomes [*F*_(3_,_1215)_ = 5.983; *p* = 0.001]. Psychology students’ group achieved the lowest mean score and significantly differed from other analyzed student groups’ results in terms of conscientiousness [*F*_(3_,_1216)_ = 8.287; *p* = 0.001]. Moreover, mean emotional stability was lower in psychology and pedagogy students’ reports when compared to medical students’ outcomes [*F*_(3_,_1215)_ = 6.628; *p* = 0.001]. The highest openness to experience was observed in psychology students’ results while students of medicine had significantly lower scores [*F*_(3_,_1213)_ = 3.615; *p* = 0.013].

### Analysis of Mean Scores

In terms of the perceived level of stress declared by medical field (medical and nursing) and social field (psychology and pedagogy) students, there was no significant difference between results in the two groups (*t* = −0.137; *p* = 0.89).

## Discussion

The aim of our study was to examine the relationship between the level of stress and QoL in a group of students from varying fields, explore personality traits as a potentially important factor in choosing university fields, and verify whether medical students in comparison to non–medical students will declare higher stress levels.

The expected relationship between higher stress levels and lower QoL was confirmed (H1 substantiated). The obtained results also highlighted personality differences between students from the four groups (H2 substantiated) with medical students being the most agreeable differing significantly from nursing and pedagogy students, and more emotionally stable than psychology and pedagogy students. Psychology students, on the other hand, were less extraverted than nursing and pedagogy students, less conscientious than all other students, and more open to experience than medical students.

Numerous researches reported the negative relationship between stress and QoL in university students (Ribeiro et al., 2018). “QoL was defined, therefore, as individuals’ perception of their position in life in the context of the culture and value systems in which they live, and in relation to their goals, expectations, standards, and concerns” (WHOQoL Group, 1995). There is also a varying outcome when comparing medical students to the general population. Some results present that undergraduate students of medicine report lower QoL level (Pagnin and De Queiroz, 2015), and other show there is no difference in QoL of all university students–however, its level is lower in students group than in a reference group (Henning et al., 2012). Regarding stress levels, we did not discover medical field students to declare higher subjective distress compared to social field students (H3 unsubstantiated).

Apart from the fact that the perceived level of stress is influenced by environmental factors, including potentially the type of study (but our study did not confirm this relationship), personality can also play a role when it comes to choosing a field of study (Pringle et al., 2010). The study carried out by Humburg suggests that personality traits are as important as cognitive skills when it comes to choosing a university field. For example, conscientiousness is positively related to the probability of studying medical sciences (Humburg, 2017). On the contrary Usslepp et al. (2020) assumed that The Big Five traits showed no or only small significant associations with educational track choices and indicated the need of understanding of the importance of various aspects of personality in affecting and structuring the lives of young adults. In our study, we discovered that there were some dependencies between personality factors and type of university fields, but it seems necessary to carry out further studies for more certain conclusions.

The presented research has a few apparent strengths, but we are also aware of its limitations. A noticeable advantage is the design of the study including four different courses from two larger fields (medical and social) together with a large sample formed by students from all years in a course. On the other hand, we used convenience sampling, reaching out to students attending a selected class, which accounted for a slight reduction of sample size and may have had an effect on the overall obtained results. Overrepresentation of women in three out of four examined fields (nursing, pedagogy, and psychology) could have affected the obtained results regarding personality traits (Costa et al., 2001; Schmitt et al., 2008) but at the same time it reflects the true structure of the students populations in aforementioned fields. Utilizing a correlational analysis restrained us from formulating any cause-and-effect conclusions.

Based on official statistics of Medical University of Gdańsk, in 2019 the general population of medical students was 1864 people, and of nursing students–414 people (Sobczak et al., 2021). Therefore, our sample accounts for 22.1% of all medical students and 60.1% nursing students form this university. In the same year, the population of psychology students at University of Gdańsk was 643, with our sample exploring 50.6% of students. For pedagogy the numbers are 889 and 26.3% (Sobczak et al., 2021). Taking into account both the sample size and its diversity in terms of people from various fields of study–both medical and non-medical–it seems that the study has a high external validity and its results can be generalized to other populations. Additional advantage is the use of standardized and reliable tools, which makes it possible to replicate the study in other countries.

Furthermore, we would like to put forward some practical implications. Firstly, the study highlights the grave importance of mental health education of students from demanding courses who often experience distress and should be taught effective coping strategies. Secondly, the popularization of mindfulness among young adults could also be beneficial for their subjective QoL (De Vibe et al., 2013). Moreover, it is worth encouraging students to look for various methods of coping with stress, which may include: listening to music, praying, yoga meditation, pursuing their hobbies, or practicing sports (Yikealo et al., 2018). Once they find the best way to minimize their stress level, they should execute it regularly. Providing better financial security, more precise goals, expectations, educational, and research requirements can also help students reduce stress and prevent future mental illness. Additionally, more effective time-out of tasks, credits, and exams preventing accumulation at the end of the year could be beneficial (Bacchi and Licinio, 2017). It also seems crucial to provide medical and social sciences students with universal and free psychological assistance, preferably by establishing university psychological centers or cooperating with external units providing such services.

In conclusion, many students are subjected to high levels of stress lowering their QoL and possibly affecting their academic performance and overall studying experience. Both medical and social field students experience this heightened distress, despite distinct public views of the mentioned fields. Students selecting different courses as their future career paths significantly differ from each other in terms of personality traits. In terms of extraversion and conscientiousness, psychology students scored lower and significantly deviated from the results of other studied groups. On the other hand, this group of students showed the highest openness to experience. Medical students presented the highest scores in terms of agreeability, which ranged significantly from nursing and pedagogy students’ results. Furthermore, when compared to medical students, both psychology and pedagogy students reported lower emotional stability. The causes for these dependencies require clarification and deeper analysis in future research. Literature on the subject, used for creating the base for the hypotheses explored in the study, and data derived from it, suggests that results of this study could be replicated in other countries and foreign populations of medical and social field students. This proves that the results of our research can be useful for preparing personalized treatment programs for students of various fields, and in caring for mental well-being during their academic years. The study highlights key traits of students from particular faculties, essential for keeping the quality of work and learning sufficiently high. Therefore, future research should focus on testing various help and treatment programs to emerge the most optimal coping strategies for students with different personality traits.

## Data Availability Statement

The raw data supporting the conclusions of this article will be made available by the authors, without undue reservation.

## Ethics Statement

The studies involving human participants were reviewed and approved by Ethics Committee at the Institute of Psychology. The patients/participants provided their written informed consent to participate in this study.

## Author Contributions

KS, AR, and AZ-R contributed to conception and design of the study. KS organized the database. MW and KG performed the statistical analysis. MW, JG, KG, EN, and KK wrote sections of the manuscript. MW, JG, and KS did the final editing. All authors contributed to the article and approved the submitted version.

## Conflict of Interest

The authors declare that the research was conducted in the absence of any commercial or financial relationships that could be construed as a potential conflict of interest.

## Publisher’s Note

All claims expressed in this article are solely those of the authors and do not necessarily represent those of their affiliated organizations, or those of the publisher, the editors and the reviewers. Any product that may be evaluated in this article, or claim that may be made by its manufacturer, is not guaranteed or endorsed by the publisher.
